# An Information-Geometric Formulation of Pattern Separation and Evaluation of Existing Indices

**DOI:** 10.3390/e26090737

**Published:** 2024-08-29

**Authors:** Harvey Wang, Selena Singh, Thomas Trappenberg, Abraham Nunes

**Affiliations:** 1 Faculty of Computer Science, Dalhousie University, Halifax, NS B3H 4R2, Canada; harvey@dal.ca (H.W.); tt@cs.dal.ca (T.T.); 2 Department of Psychology, Neuroscience & Behaviour, McMaster University, Hamilton, ON L8S 4L8, Canada; singhs11@mcmaster.ca; 3 Department of Psychiatry, Dalhousie University, Halifax, NS B3H 4R2, Canada

**Keywords:** pattern separation, information geometry, information theory

## Abstract

Pattern separation is a computational process by which dissimilar neural patterns are generated from similar input patterns. We present an information-geometric formulation of pattern separation, where a pattern separator is modeled as a family of statistical distributions on a manifold. Such a manifold maps an input (i.e., coordinates) to a probability distribution that generates firing patterns. Pattern separation occurs when small coordinate changes result in large distances between samples from the corresponding distributions. Under this formulation, we implement a two-neuron system whose probability law forms a three-dimensional manifold with mutually orthogonal coordinates representing the neurons’ marginal and correlational firing rates. We use this highly controlled system to examine the behavior of spike train similarity indices commonly used in pattern separation research. We find that all indices (except scaling factor) are sensitive to relative differences in marginal firing rates, but no index adequately captures differences in spike trains that result from altering the correlation in activity between the two neurons. That is, existing pattern separation metrics appear (A) sensitive to patterns that are encoded by different neurons but (B) insensitive to patterns that differ only in relative spike timing (e.g., synchrony between neurons in the ensemble).

## 1. Introduction

### 1.1. Pattern Separation in Computational Neuroscience

The hippocampus plays a key role in the formation of complex associative and episodic memories [1,2]. Classical computational models have proposed that the hippocampus performs two complementary neural computations to minimize interference and maximize information storage: *pattern separation* and *pattern completion* [3]. Pattern separation is a computation performed by a neural network that minimizes the similarity between distinct but overlapping input patterns [4]. It is thought to be performed prior to long-term memory storage to reduce the probability of interference in memory recall and enhance downstream pattern completion [5]. Pattern completion, in contrast to pattern separation, is performed by a network during the retrieval of stored patterns when presented with partial or degraded input patterns.

The hippocampal dentate gyrus has a set of properties that makes it ideally suited to perform pattern separation, such as the sparse and competitive firing of granule cells [6,7]. Rodent studies have shown that activity patterns in the dentate gyrus are less correlated than those in the entorhinal cortex and hippocampal CA3 region, consistent with dentate gyrus pattern separation [8]. Importantly, pattern separation is not only found in the hippocampus. The cerebellum and insect mushroom body also have properties that would facilitate pattern separation [9]. Thus, pattern separation is a fundamental computation utilized by the brain to reduce interference between activity patterns to promote functions such as memory encoding.

Physiological changes in the dentate gyrus have been linked to conditions involving cognitive impairments, including schizophrenia [10], Alzheimer’s disease [11], and epilepsy [12]. Patients affected by these conditions present with reduced abilities to distinguish between similar stimuli [13,14,15]. There is, therefore, great interest in the detailed biophysical mechanisms underlying pattern separation. It has been theorized that ensembles of interconnected, transiently active neurons encode and transfer information with their precise timing in neural firing [16], thus enabling numerous operations in the brain [17,18]. Many computational models of such neuron assemblies have been implemented to simulate pattern separation under various physiological conditions [19,20,21,22]. However, when studied in isolation, “pattern separation” is often defined even more arbitrarily, often reduced to refer to any computation that minimizes similarity or overlap (see further discussions in Section 1.2 and Section 3), and many of the aforementioned studies often employ inconsistent measurements for it. How do we simulate a computation for which there is no precise definition? Such definition, therefore, is essential to further the understanding of what pattern separation is and is not, as well as providing a theoretical basis for further studies.

### 1.2. There Exists No Single Definition for Pattern Separation

Pattern separation is generally defined as a computation that maximizes *dissimilarity* or *orthogonalization* [23,24] between neural patterns, given similar yet distinct input patterns [25,26,27]. Definition 1 formalizes this concept.

**Definition** **1.** *For some dissimilarity or distance measure d and input neural patterns $X^{\left(1\right)}$ and $X^{\left(2\right)}$, the mapping f performs pattern separation if and only if the dissimilarity between its outputs is greater than that between $X^{\left(1\right)}$ and $X^{\left(2\right)}$:*

$$d\left(X^{\left(1\right)},X^{\left(2\right)}\right)<d\left(f\left(X^{\left(1\right)}\right),f\left(X^{\left(2\right)}\right)\right).$$

Clearly, the definition of pattern separation relies heavily on the form of the dissimilarity measure *d*. Definition 1 is in fact not a definition for a single, but rather, for multiple computations. This creates a problem where different forms of *d* would translate to different interpretations for pattern separation. For example, if Pearson correlation is used for *d*, then pattern separation would be a computation that minimizes *correlation*; if *d* is the Hamming distance, then pattern separation is a computation that minimizes *overlap*.

Having a diverse set of indices, all describing “pattern separation”, limits the comparability of pattern separation efficacy across computational studies [20,23,28]. Madar et al. [29] showed that quantifying the pattern separation abilities of a system by applying different measures *d* to the same dataset lead to different conclusions; for example, they showed that pairs of spike trains can be uncorrelated without being orthogonal, and therefore suggested that pattern separation should be considered *a group of potential computations*, since it is unclear which spike train features are most relevant to the brain and what constitutes similarity or dissimilarity. For instance, if information was coded using a neuron’s firing rate, a pattern separator would aim to convert a series of input trains with similar rates to ones with dissimilar rates. On the other hand, the firing rate could carry no information, and the system would rather use spike timing (or temporal coding) to represent information; in this case, the pattern separator may aim to simply change the relative timing of spike trains. A recent analysis of the most commonly used pattern separation indices demonstrated that some failed to capture information loss that may occur with high degrees of sparsity (a property often associated with strong pattern separation), and instead found that these measures confounded information loss with excellent pattern separation [30]. Although these studies evaluated existing pattern separation measures, the evaluations were not conducted in such a way that would control for degrees of separation achieved by different pattern separation strategies. Taken together, these analyses of the most commonly used pattern separation measures highlight major issues with consistency, measurement accuracy, and applicability between studies, and highlight the importance of developing a single, unifying definition of pattern separation, along with a controlled re-evaluation of existing measures.

### 1.3. An Information-Geometric Formulation of Pattern Separation

In this work, we use tools from information geometry to describe pattern separation as a *single* computation. This formulation is consistent with the existing notion of pattern separation (Definition 1) but also addresses shortcomings of existing definitions and measures.

A pattern separator (e.g., a neural network such as the hippocampal dentate gyrus) and its inputs (e.g., from the entorhinal cortex) are respectively modeled as a statistical manifold and its coordinates. A point on this manifold is a probability distribution that (A) is identified by its input coordinates and (B) generates output patterns (i.e., spike trains) as samples. A highly effective separator maps input patterns with small differences to output patterns with large differences. That is, small differences between the input spike trains representing different patterns (which here constitute small changes in coordinates), would result in very different output spike trains from the neural pattern separator. Under our formulation, an excellent pattern separator would correspond to a statistical manifold with high curvature that maps small changes in its coordinates to distributions whose samples are highly distant or distinct from each other. In such manifolds, a single sample from a distribution provides a large degree of information about the underlying parameters or inputs by which it was generated. This is not only consistent with traditional notions of pattern separation but also satisfies the problem identified by Bird et al. [30], where common measures can fail to distinguish between pattern separation and information loss (which could occur by randomly adding noise to a pattern).

We demonstrate an application of this formalism by implementing a two-neuron system with an analytically tractable probability law such that distances between spike train distributions on the underlying manifold can be controlled. Using this system, we can simulate pattern separation that occurs by (A) changing *which neurons* are active across patterns, (B) changing *how much* the neurons are active, and (C) changing the *relative timing* of neural spikes across patterns. We then evaluate the degree to which several commonly used pattern separation indices are sensitive to each of these changes. These three circuit properties ultimately sculpt the types of spatiotemporal firing patterns that a circuit is ideally suited to produce and process [16]. Specific groups of neurons may have distinct combinations of the above three properties to facilitate the same neural computation repeatedly [16], which may serve to ensure efficient and reliable neural computation. Precise spike timing has been theorized to underlie information transfer within and between neuronal ensembles [18], and repeating connectivity patterns between these ensembles across the neocortex may additionally facilitate spike-timing-dependent neural computation, localized to specific cortical regions [31]. Therefore, the three parameters we choose here are relevant to known mechanisms of pattern generation and encoding in the brain, and we simulate pattern separation by subsequently modulating these parameters.

### 1.4. An Evaluation of Existing Indices of Pattern Separation

Existing indices appear much more sensitive to pattern separation as a result of changes in the neuron’s firing rates, and appear, in general, insensitive to pattern separation as a result of changes in the rate of the neuron’s coincident firing (and thus the relatively timing by which the neurons are active). In addition, our results provide further evidence that, while sensitive to changes in neuronal firing rates, the variety of existing pattern separation indices could lead to inconsistent conclusions on the amount of pattern separation performed.

## 2. An Information-Geometric Formulation of Pattern Separation

### 2.1. Pattern Separator Described by a Statistical Manifold

Let us use an *N*-dimensional statistical manifold P to describe the neural activity of a pattern separator, such as the hippocampal dentate gyrus, in response to different inputs. Consider the manifold’s coordinates ξ, representing parameters that influence the separator’s outputs. These parameters can be subdivided into two different types: (A) those that are intrinsic to the system, such as network connectivity and other biophysical properties, and (B) those received as input patterns from other areas, such as the perforant path inputs from entorhinal cortex to the hippocampal dentate gyrus. Each distinct parameterization ξ specifies a probability distribution p(x;ξ) on P, from which neural patterns x may be drawn. In the case that the intrinsic parameters are fixed, the change in coordinates $d\xi ={\left(d\xi_{i}\right)}_{i=1,\ldots ,N}$ describes the change in the inputs, and the distance between the probability distributions reflects the change in the outputs.

A highly effective pattern separator maps input patterns with small differences to output patterns with large differences. Under our information-geometric formulation, this would correspond to a manifold P with high curvature, which maps small changes in its coordinates dξ, to distributions whose samples are highly distant or distinct from each other, p(x;ξ) and p(x;ξ+dξ). Under this model, a single output sample provides a large degree of information about the underlying parameters by which it was generated.

The definition of distance between probability distributions p(x;ξ) and p(x;ξ+dξ) is given by the *Fisher information matrix*. The direction and magnitude of change in a probability distribution, as a result of a small change $d\xi_{i}$ in the $i^{\mathrm{th}}$ coordinate, are described by the derivative with respect to $d\xi_{i}$,

$$L\left(\xi_{i}\right)=\frac{\partial \log p(x;\xi )}{\partial \xi_{i}},$$

where L is known as the score function. Elements of the Fisher information matrix $G={\left(g(\xi_{i},\xi_{j})\right)}_{i,j=1,\ldots ,N}$ are given by the expectation of the product of the score functions for two coordinates

(1) $$\begin{array}{cc} g(\xi_{i},\xi_{j})\; & ={\langle L\left(\xi_{i}\right)L\left(\xi_{j}\right)\rangle }_{x}\\ & ={\langle \frac{\partial \log p(x;\xi )}{\partial \xi_{i}}\frac{\partial \log p(x;\xi )}{\partial \xi_{j}}\rangle }_{x}, \end{array}$$

where ${\langle f(x)\rangle }_{x}$ denotes the expectation value of a function f(x), defined as the weighted average over all possible values of x, $\sum_{x}f\left(x\right)p\left(x\right)$. The squared distance between two probability distributions defined by ξ and ξ+dξ can then be computed by the quadratic form of dξ,

(2) $$ds^{2}=\sum_{i=1}^{N}\sum_{j=1}^{N}g\left(\xi_{i},\xi_{j}\right)\;d\xi_{i}d\xi_{j}.$$

In our formulation of pattern separation, two different coordinates, ξ and ξ+dξ, represent two different input patterns. These coordinates define two different probability distributions, p(x;ξ) and p(x;ξ+dξ), whose samples are less overlapping, or more separated, as a result of dξ. This conceptualization of pattern separation is consistent with its existing notion given in Definition 1, and provides a clear mathematical formalism that we demonstrate when analyzing different pattern separation metrics.

It is important to note that while we can *directly* simulate changes dξ theoretically, ξ and dξ are only *indirectly* influenced in real neural systems. For example, pattern separation may be achieved by drastically altering intrinsic parameters such as network connectivity and neuronal biophysics. We emphasize here that these properties may change within biological systems and affect pattern separation performance, but these changes occur over longer time scales than what is most relevant for pattern separation of two similar and highly overlapping stimuli presented consecutively. We, however, still consider these mechanisms of achieving pattern separation. As such, to evaluate the existing indices, we manipulate dξ to simulate changes in the input patterns or physiological properties of the network, giving us a ground truth for the *type* and *degree* of the simulated pattern separation.

### 2.2. A Two-Neuron Manifold and Its Orthogonal Coordinates

Consider a model of a system of two neurons [32] and its time-independent firing pattern $x={\left(x_{i}\right)}_{i=1,2}$ in a given observation window, where $x_{i}=1$ and $x_{i}=0$ indicate that the *i*th neuron is active (with one or more spikes) or silent, respectively. We use the highly controlled probability distribution p(x;ξ) for this model to separately alter the independent firing rates and the degree of correlation between neurons. Under the formalism introduced above, these manipulations change the coordinates on the system’s manifold. Controlling the amount of pattern separation, as well as the strategy by which it is achieved, facilitates our evaluation of the existing indices. Specifically, we can use this model to simulate the pattern separation that arises from changes in each neuron’s independent firing rates, the relative timing that they are active, *or* both.

The probability distribution p(x;ξ) for this model is given by the probabilities that each neuron is active or silent,

$$q_{mn}=\mathrm{Prob}\left\{x_{1}=m,\;x_{2}=n\right\},\;m,n=0,1.$$

These probabilities give an exact expansion of the logarithm of the probability distribution of the system,

$$\log p\left(x;\xi \right)=\log\frac{q_{10}}{q_{00}}x_{1}+\log\frac{q_{01}}{q_{00}}x_{2}+\theta x_{1}x_{2}+\log q_{00},$$

where

(3) $$\theta =\log\frac{q_{11}q_{00}}{q_{10}q_{01}}.$$

The term θ specifies the within-ensemble correlation that represents the degree to which neurons 1 and 2 are correlated. θ is 0 when the two neurons are uncorrelated, approaches −∞ as the two neurons become *maximally anti-correlated* (as $q_{11}$ and $q_{00}$ approach 0), and approaches *∞* as the two neurons become *maximally correlated* (as $q_{10}$ and $q_{01}$ approach 1).

Among the set of four probabilities, $\left\{q_{00},q_{10},q_{01},q_{11}\right\}$, only three variables are free, due to the constraint $q_{00}+q_{10}+q_{01}+q_{11}=1.$ The manifold for this two-neuron model is therefore three dimensional. Though there are many coordinate systems for this model (for example, any of the three from $\left\{q_{00},q_{10},q_{01},q_{11}\right\}$), we use

$$\xi =(\eta_{1},\eta_{2},\theta )$$

to simulate different types of pattern separation, where the variables

(4) $$\begin{array}{c} \eta_{1}=\langle x_{1}\rangle =q_{10}+q_{11},\;\eta_{2}=\langle x_{2}\rangle =q_{01}+q_{11}, \end{array}$$

denote the marginal firing rates of neurons 1 and 2, respectively. Changes in $\eta_{1},\eta_{2},\theta$ represent the means by which pattern separation may be achieved in a neural system in equilibrium: by changing (A) *which neurons* fire, (B) *how much* they fire, and/or (C) the *relative timing* at which they fire.

The parameters $\eta_{1},\eta_{2},\theta$ form a convenient coordinate system because $\left(\eta_{1},\eta_{2}\right)$ and θ are mutually orthogonal, meaning that their directions of change are uncorrelated [32]. Specifically, this means that

(5) $$g\left(\eta_{1},\theta \right)=g\left(\eta_{2},\theta \right)=0.$$

Crucially, the orthogonality between $\left(\eta_{1},\eta_{2}\right)$ and θ allows us to increase the distance between two probability distributions by changing *only* dθ *or* one of $d\eta_{1}$ and $d\eta_{2}$. In other words, for two distributions with the same values of $\eta_{1}$ and $\eta_{2}$, the distance between them is proportional to the difference between their θ values,

(6) $$ds^{2}{|}_{d\eta_{1}=d\eta_{2}=0}=g\left(\theta ,\theta \right)\;{\left(d\theta \right)}^{2}.$$

Similarly, this orthogonality simplifies Equation (2) in the case where dθ=0

(7) $$ds^{2}{|}_{d\theta =0}=2\;g\left(\eta_{1},\eta_{2}\right)\;d\eta_{1}d\eta_{2}+g\left(\eta_{1},\eta_{1}\right)\;{\left(d\eta_{1}\right)}^{2}+g\left(\eta_{2},\eta_{2}\right)\;{\left(d\eta_{2}\right)}^{2}.$$

Using this setup, we define a “control” system by specifying its probability distribution using these three variables, denoted as $p(x;\eta_{1},\eta_{2},\theta )$. Using similar notation, we define a second “comparison” distribution $p(x;\eta_{1}+d\eta_{1},\eta_{2}+d\eta_{2},\theta +d\theta )$, and thus simulate pattern separation by systematically manipulating the values of $d\eta_{1}$, $d\eta_{2}$, and dθ to alter the distance between $p(x;\eta_{1},\eta_{2},\theta )$ and $p(x;\eta_{1}+d\eta_{1},\eta_{2}+d\eta_{2},\theta +d\theta )$. The changes in the parameters of the two distributions (i.e., $d\eta_{1}$, $d\eta_{2}$, and dθ) represent the differences between two input patterns presented to a pattern separator, and the distance between the distributions represents the differences between the corresponding outputs from the separator. Pattern separation is therefore achieved in this system when small $d\eta_{1}$, $d\eta_{2}$, and/or dθ result in a large distance between the control and comparison distributions.

An appropriate index of pattern separation should be sensitive to the increased distance between $p(x;\eta_{1},\eta_{2},\theta )$ and $p(x;\eta_{1}+d\eta_{1},\eta_{2}+d\eta_{2},\theta +d\theta )$. This increased distance corresponds to pattern separation as a result of changes in each neuron’s firing rates, relative timing of activity, *or* a combination of both.

### 2.3. Generating Diverging Patterns

Neural patterns were generated from control and comparison distributions $p(x;\eta_{1},\eta_{2},\theta )$ and $p(x;\eta_{1}+d\eta_{1},\eta_{2}+d\eta_{2},\theta +d\theta )$, by sampling $N_{\mathrm{bins}}=10^{3}$ times from each distribution. Each sample constituted a “time bin” in which one, both, or neither of the neurons may be active; that is, samples were drawn from categorical distributions with event probabilities $\left\{q_{00},q_{10},q_{01},q_{11}\right\}$ for each time bin.

#### 2.3.1. Patterns with Dissimilar Neuronal Coincident Firing Rates

To assess the sensitivity of existing indices in detecting pattern separation as a result of changes in within-ensemble, neuron–neuron correlation structures, we set the marginal firing rates of the two neurons constant and gradually increased dθ. Specifically, we set the control distribution to $p(x;\eta_{1},\eta_{2},0)$, and the comparison to $p(x;\eta_{1},\eta_{2},d\theta )$. Recall from Equation (6), the squared distance between the two distributions is proportional to ${\left(d\theta \right)}^{2}$ in this setup.

We assigned new values for θ by changing $q_{11}$ and $q_{00}$ the same amount in the same direction, while changing $q_{10}$ and $q_{01}$ the same amount in the opposite direction, thereby keeping $\eta_{1}$ and $\eta_{2}$ constant. In other words, $q_{00},q_{10},q_{01},\mathrm{and}\;q_{11}$ were updated by the following

$$\begin{array}{c} q_{00}\leftarrow q_{00}+dq_{11},\;q_{10}\leftarrow q_{10}-dq_{11},\;q_{01}\leftarrow q_{01}-dq_{11},\;q_{11}\leftarrow q_{11}+dq_{11},\; \end{array}$$

so that the corresponding new θ was

$$\begin{array}{c} \theta \leftarrow \log\frac{\left(q_{11}+dq_{11}\right)\left(q_{00}+dq_{11}\right)}{\left(q_{10}-dq_{11}\right)\left(q_{01}-dq_{11}\right)}, \end{array}$$

where $\eta_{1}$ and $\eta_{2}$ remained the same

$$\begin{array}{c} \eta_{1}\leftarrow \left(q_{10}-dq_{11}\right)+\left(q_{11}+dq_{11}\right)=q_{10}+q_{11},\\ \eta_{2}\leftarrow \left(q_{01}-dq_{11}\right)+\left(q_{11}+dq_{11}\right)=q_{01}+q_{11}. \end{array}$$

This procedure was repeated for different values of $\eta_{1}$ and $\eta_{2}\geq \eta_{1}$. We also applied the additional constraints $\eta_{2}=1-\eta_{1}$ to keep the overall firing rate of the system constant.

Overall, this experimental setup created comparison distributions whose two neurons ranged from being nearly maximally anti-correlated (when $q_{11}=q_{00}=10^{-2}$ so that dθ is small) to nearly maximally correlated (when $q_{10}=10^{-2}$ so that dθ is large).

#### 2.3.2. Patterns with Dissimilar Neuronal Firing Rates

To assess the sensitivity of existing indices in detecting pattern separation as a result of changes in one neuron’s firing rate, we set the marginal firing rate of one of the neurons to be constant, allowed the neurons to fire independently, and gradually increased $d\eta_{2}$. Specifically, we set the control distribution to $p(x;\eta_{1},0.1,0)$ and the comparison to $p(x;\eta_{1},0.1+d\eta_{2},0)$. The squared distance between these two distributions is proportional to ${\left(d\eta_{2}\right)}^{2}$:

(8) $$ds^{2}{|}_{d\eta_{1}=d\theta =0}=g\left(\eta_{2},\eta_{2}\right)\;{\left(d\eta_{2}\right)}^{2}.$$

We defined our systems by expressing $\left\{q_{00},q_{10},q_{01},q_{11}\right\}$ as functions of $\eta_{1}$ and $\eta_{2}$ at θ=0. Since θ=0 means that neurons 1 and 2 fire independently, $q_{11}=\eta_{1}\eta_{2}$. We could also, of course, show this by directly using the two-neuron model, with

$$\begin{array}{cc} \theta =\log\frac{q_{11}q_{00}}{q_{10}q_{01}}=0 & \Leftrightarrow \frac{q_{11}q_{00}}{q_{10}q_{01}}=1\Leftrightarrow q_{11}=\frac{q_{10}q_{01}}{q_{00}}. \end{array}$$

Since $q_{10}=\eta_{1}-q_{11}$, $q_{01}=\eta_{2}-q_{11}$, and $q_{00}=1-q_{10}-q_{01}-q_{11}$, we can rearrange the above,

$$\begin{array}{cc} q_{11}\; & =\frac{\left(\eta_{1}-q_{11}\right)\left(\eta_{2}-q_{11}\right)}{1-\left(\eta_{1}-q_{11}\right)-\left(\eta_{2}-q_{11}\right)-q_{11}}\\ & \Leftrightarrow \left(\eta_{1}-1\right)\left(\eta_{2}-1\right)=1-\eta_{1}-\eta_{2}+q_{11}\\ & \Leftrightarrow q_{11}=\eta_{1}\eta_{2}. \end{array}$$

Overall, this experimental setup created comparison distributions that permitted one of the neurons to range from sparsely active (when $d\eta_{2}$ was small) to densely firing (when $d\eta_{2}$ was large).

## 3. Existing Indices

Recall from Definition 1 that pattern separation is characterized by the increase in pairwise dissimilarity between two neural patterns. Specifically, the mapping *f* performs pattern separation if and only if,

$$d\left(X^{\left(1\right)},X^{\left(2\right)}\right)<d\left(f\left(X^{\left(1\right)}\right),f\left(X^{\left(2\right)}\right)\right),$$

where *d* is some index of dissimilarity between two neural patterns $X^{\left(1\right)}$ and $X^{\left(2\right)}$. The superscripts (1) and (2) respectively denote the first and second patterns examined. Individual indices are denoted as *d* with subscripts.

### 3.1. Pattern Representation

Existing indices of pattern separation operate on one of two representations of neural patterns: discretized vectors x or non-discretized lists *T* (the notation X above merely denotes the pattern as a random variable but makes no specification on how the pattern is represented).

#### 3.1.1. Non-Discretized Patterns

Consider a system of $N_{\mathrm{neurons}}$ neurons. The spiking pattern of this system can be represented as a list of spike trains from each individual neuron $T={\left\{T_{i}\right\}}_{i=1,\ldots ,N_{\mathrm{neurons}}}$. $T_{i}={\left\{T_{ij}\right\}}_{j=1,\ldots ,n_{i}}$ denotes the list of spike times $T_{ij}$ from the ith neuron, where $n_{i}$ is the total number of times the neuron fires.

#### 3.1.2. Discretized Patterns

A pattern *T* can be binned and binarized so that a wider variety of indices can be applied. This is generally performed by dividing the observation window into $N_{\mathrm{bins}}$ equally sized bins, and assigning each bin a binary value $x_{ij}\in \left\{0,1\right\}$. $x_{ij}=1$ indicates that the ith neuron is active at least once in the jth bin, and $x_{ij}=0$ otherwise. The matrix representation of a binarized pattern is then simply $X={\left(x_{ij}\right)}_{i=1,\ldots ,N_{\mathrm{neurons}}}^{j=1,\ldots ,N_{\mathrm{bins}}}$. These matrices are then generally vectorized into

(9) $$\begin{array}{cc} x\; & =\mathrm{vec}(X^{⊺})\\ \; & =[x_{11},x_{12},\ldots ,x_{1N_{\mathrm{bins}}},x_{21},x_{22},\ldots ,x_{2N_{\mathrm{bins}}},\ldots x_{N_{\mathrm{neurons}}1},x_{N_{\mathrm{neurons}}2},\ldots ,x_{N_{\mathrm{neurons}}N_{\mathrm{bins}}}]. \end{array}$$

### 3.2. Common Indices

The **Pearson correlation**,

(10) $$d_{\rho }\left(x^{\left(1\right)},x^{\left(2\right)}\right)=\frac{\langle x^{\left(1\right)}x^{\left(2\right)}\rangle -\langle x^{\left(1\right)}\rangle \langle x^{\left(2\right)}\rangle }{\sqrt{\langle {\left(x^{\left(1\right)}\right)}^{2}\rangle -{\langle x^{\left(1\right)}\rangle }^{2}}\sqrt{\langle {\left(x^{\left(2\right)}\right)}^{2}\rangle -{\langle x^{\left(2\right)}\rangle }^{2}}},$$

is one of the most widely used indices. It has been used to study pattern separation both in computational [33] and experimental studies [8,34,35,36], as well as under various physiological conditions, such as during epileptic hyperexcitability in the dentate gyrus [33].

The **cosine similarity**, or the normalized dot product,

(11) $$d_{\theta }\left(x^{\left(1\right)},x^{\left(2\right)}\right)=\frac{x^{\left(1\right)}\cdot x^{\left(2\right)}}{∥x^{\left(1\right)}∥∥x^{\left(2\right)}∥},$$

is another popular index of pattern separation. The original Hebb–Marr framework theorized pattern separation as the orthogonalization of the input patterns [23,24]. The terms “decorrelation” (as described by the Pearson correlation) and “orthogonalization”, however, are not mathematically equivalent; Madar et al. [29] explicitly showed that pairs of spike trains can be uncorrelated without being orthogonal, or can be orthogonal without being uncorrelated. The cosine similarity has thus been used to explicitly determine whether spike trains are truly orthogonalized.

While the cosine similarity measures the angle between two vector representations of spike trains, the **scaling factor**,

(12) $$d_{\phi }\left(x^{\left(1\right)},x^{\left(2\right)}\right)=\frac{∥x^{\left(1\right)}∥}{∥x^{\left(2\right)}∥},$$

quantifies their difference in norm. Together, $d_{\theta }$ and $d_{\phi }$ are, in theory, sufficient to fully describe the similarity between two vectors in Euclidean space [29] since they focus on complementary features, angle and norm.

One implementation of the **Hamming distance**, or population distance, between two neural patterns, is given by

$$d_{f}=\frac{\mathrm{HD}}{2N(1-s)},$$

where HD denotes the number of positions at which the corresponding values are different [37], and *s* the sparsity. $d_{f}$ is used to evaluate pattern separation in several papers [19,20,38]. This implementation of Hamming distance is limited by its lack of consideration of the temporal aspect of a given pattern—a cell is considered active if it fires at least once during stimulus presentation, regardless of time and frequency [38]. We therefore use a modified definition of the Hamming distance [30], and take the average of the absolute difference at each time bin,

(13) $$d_{\eta }\left(x^{\left(1\right)},x^{\left(2\right)}\right)=\frac{1}{N_{\mathrm{neurons}}\;N_{\mathrm{bins}}}\sum_{i=1}^{N_{\mathrm{neurons}}}\sum_{j=1}^{N_{\mathrm{bins}}}\left|x_{ij}^{\left(1\right)}-x_{ij}^{\left(2\right)}\right|,$$

where permanently inactive spike trains are not removed from the ensemble, and the bins in which each neuron spikes are taken into consideration.

The **SPIKE similarity** was designed to assess the dissimilarity between two spike trains [39]. We compute the SPIKE similarity between two neural patterns, collected from an ensemble of neurons, as the average across each neuron,

(14) $$d_{S}\left(T^{\left(1\right)},T^{\left(2\right)}\right)=\frac{1}{N_{\mathrm{neurons}}}\sum_{i=1}^{N_{\mathrm{neurons}}}\delta_{S}\left(T_{i}^{\left(1\right)},T_{i}^{\left(2\right)}\right).$$

The quantity

$$\delta_{S}\left(T_{i}^{\left(1\right)},T_{i}^{\left(2\right)}\right)=1-\frac{1}{\tau }\int_{0}^{\tau }dt\;D\left(t\right),$$

is used to assess the dissimilarity between two spike trains [29]. It is computed by integrating, over the observation window [0,τ], the distance between two spike trains from Kreuz et al. [39],

$$D\left(t\right)=\frac{S^{\left(1\right)}x_{\mathrm{ISI}}^{\left(2\right)}+S^{\left(2\right)}x_{\mathrm{ISI}}^{\left(1\right)}}{2{\langle x_{\mathrm{ISI}}^{\left(n\right)}\rangle }^{2}},$$

where

$$S^{\left(n\right)}\left(t\right)=\frac{d{t_{P}}^{\left(n\right)}x_{F}^{\left(n\right)}+d{t_{F}}^{\left(n\right)}x_{P}^{\left(n\right)}}{x_{\mathrm{ISI}}^{\left(n\right)}}$$

is the local weighting for the spike time differences of each spike train.

**Figure 1:**
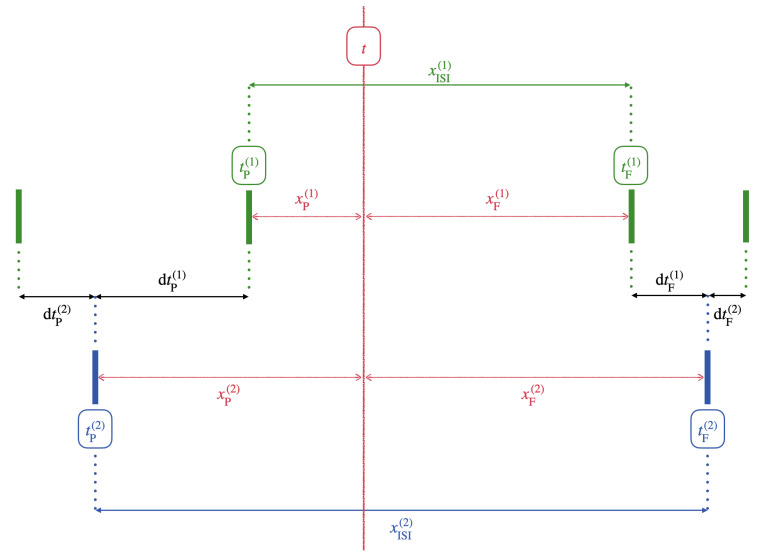
Variables used to compute the SPIKE similarity. The green and blue solid bars respectively represent the spike times of spike trains 1 and 2, relative to some observation time *t*, shown by the red line.

Figure 1 shows the variables involved in computing the SPIKE similarity. For any given time *t*, the preceding (denoted with subscript P) and following (denoted with subscript F) spike times are respectively given by

$$\begin{array}{c} {t_{P}}^{\left(n\right)}=\max_{j}\left(T_{ij}^{\left(n\right)}\;|\;T_{ij}^{\left(n\right)}\leq t\right),\;{t_{F}}^{\left(n\right)}=\min_{j}\left(T_{ij}^{\left(n\right)}\;|\;T_{ij}^{\left(n\right)}>t\right). \end{array}$$

The instantaneous inter-spike interval (denoted with subscript ISI) for each neuron is

$$x_{\mathrm{ISI}}^{\left(n\right)}={t_{F}}^{\left(n\right)}-{t_{P}}^{\left(n\right)}.$$

The intervals to the previous and following spikes for each neuron are denoted as

$$\begin{array}{c} x_{P}^{\left(n\right)}=t-{t_{P}}^{\left(n\right)},\;x_{F}^{\left(n\right)}={t_{F}}^{\left(n\right)}-t. \end{array}$$

For pattern 1, the instantaneous absolute differences of the preceding and following spike times are, respectively,

$$\begin{array}{c} d{t_{P}}^{\left(1\right)}=\min_{j}\left(\left|{t_{P}}^{\left(1\right)}-T_{ij}^{\left(2\right)}\right|\right),\;d{t_{F}}^{\left(1\right)}\left(t\right)=\min_{j}\left(\left|{t_{F}}^{\left(1\right)}-T_{ij}^{\left(2\right)}\right|\right). \end{array}$$

For pattern 2, $d{t_{P}}^{\left(2\right)}$ and $d{t_{F}}^{\left(2\right)}$ are defined analogously.

### 3.3. Information-Theoretic Measures

In addition to common indices of pattern separation found in pattern separation research, we also evaluate three information-theoretic measures using the toolbox provided by Bird et al. [30], who first applied these measures to neural patterns. These indices are used to show that common indices, described in the previous section, could conflate pattern separation with information loss.

The **estimated mutual information** ${\hat{d}}_{M}$ is computed based on the modified Kozachenko–Leonenko estimator, adopted from Houghton [40]. Briefly, patterns $T^{\left(1\right)}$ and $T^{\left(2\right)}$ are each divided into *N* periods of equal size. The pairwise spike train distances between each period of each spike train are computed using the Wasserstein distance δ (see [30,41,42]). This produces a set of pairwise distances between periods of $T^{\left(1\right)}$ and $T^{\left(2\right)}$. A biased estimator of the mutual information between $T^{\left(1\right)}$ and $T^{\left(2\right)}$, in terms of an integer smoothing parameter 1≤h<N, is given by

(15) $${\hat{d}}_{M}\left(T^{\left(1\right)},T^{\left(2\right)}\right)=\frac{1}{N}\sum_{i=1}^{N}\log\frac{N\left|C\left(T_{i}^{\left(1\right)},T_{i}^{\left(2\right)}\right)\right|}{h^{2}},$$

where $\left|C\left(T_{i}^{\left(1\right)},T_{i}^{\left(2\right)}\right)\right|$ is number of pairs $\left(T_{j}^{\left(1\right)},T_{j}^{\left(2\right)}\right)$ such that both $\delta \left(T_{i}^{\left(1\right)},T_{j}^{\left(1\right)}\right)<\delta_{h}\left(T_{i}^{\left(1\right)}\right)$ and $\delta \left(T_{i}^{\left(2\right)},T_{j}^{\left(2\right)}\right)<\delta_{h}\left(T_{i}^{\left(2\right)}\right)$, where $\delta_{h}\left(T_{i}\right)$ is the distance of $T_{i}$ to its $h^{\mathrm{th}}$ nearest neighbor amongst all segments in *T*.

The **transfer entropy** [43] from one pattern $T^{\left(1\right)}$ to another $T^{\left(2\right)}$ is the mutual information between $T^{\left(2\right)}$ at the current time τ and the history of $T^{\left(1\right)}$ conditioned on the history of $T^{\left(2\right)}$:

(16) $$d_{T}\left[T^{\left(1\right)}:T^{\left(2\right)}\right]=H\left(T_{\tau }^{\left(2\right)}|T_{t<\tau }^{\left(2\right)}\right)-H\left(T_{\tau }^{\left(2\right)}|T_{t<\tau }^{\left(2\right)},T_{t<\tau }^{\left(1\right)}\right),$$

Unlike other measures, $d_{T}$ is directional and asymmetrical (whose arguments are thus denoted using square brackets and colon [:]). In many circumstances, the transfer entropy requires less data to produce an accurate estimate than the mutual information [44,45].

The **relative redundancy reduction** is given by

(17) $$d_{R}\left(T^{\left(1\right)},T^{\left(2\right)}\right)=\left[R\left(T^{\left(1\right)}\right)-R\left(T^{\left(2\right)}\right)\right]\;{\hat{d}}_{M}\left(T^{\left(1\right)},T^{\left(2\right)}\right),$$

where R(T) is the redundancy of pattern *T*, quantifying the parts of a signal that may encode the same information. The redundancy *R* is estimated by adopting from Williams and Beer [46] to apply to pattern *T*,

$$\begin{array}{c} R\left(T\right)=\min_{T_{i}}\left[{\hat{d}}_{M}\left(T\setminus T_{i},T_{i}\right)\right]. \end{array}$$

## 4. An Evaluation of Existing Pattern Separation Indices

Table 1 shows the indices compared in this study, which are described in greater detail in the previous section. These existing indices are computed on two sample patterns, one drawn from the control distribution and the other from the comparison distribution, which are described in Section 2.3. Recall from Section 3.1 that an index *d* operates one of two representations of neural patterns: binarized vectors and undiscretized spike times.

For each measurement of an index *d*, vector $x^{\left(1\right)}$ is generated from the control distribution, and $x^{\left(2\right)}$ from the comparison distribution. Recall that each of these distributions specifies a unique set of $\left\{q_{00},q_{10},q_{01},q_{11}\right\}$, which can then be used as event probabilities for a categorical distribution, from which we sample $N_{\mathrm{bins}}$ times to construct a neural pattern x. For indices that operate on undiscretized patterns, $x^{\left(1\right)}$ and $x^{\left(2\right)}$ are converted to $T^{\left(1\right)}$ and $T^{\left(2\right)}$ by dividing a 0 to 16 ms observation period into $N_{\mathrm{bins}}$ bins, and adding time stamps that correspond to active bins into ordered sets. Each existing index is measured on $N_{\mathrm{trials}}=10$ control and comparison sample pattern pairs, then averaged. Pearson correlation, cosine similarity, scaling factor, and SPIKE similarity are reviewed by Madar et al. [29] and implemented in this study using custom Python scripts. Estimated mutual information, estimated redundancy reduction, and transfer entropy are proposed by Bird et al. [30] and computed using their Matlab toolbox. The values of $N_{\mathrm{bins}}$ and $N_{\mathrm{trials}}$ are chosen to be large enough to decrease the standard error of the mean, yet still small enough to be computationally tractable.

### 4.1. Pattern Separation via Dissimilar Within-Ensemble Correlation

**Figure 2:**
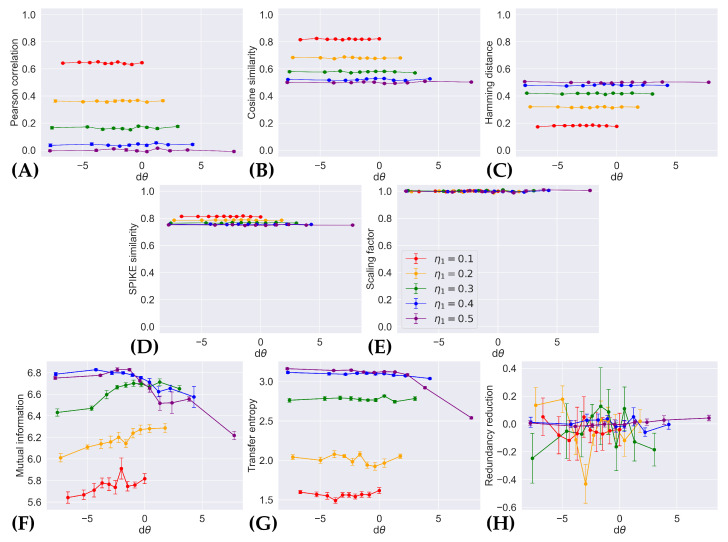
Measurements of existing pattern separation indices versus change in the rate of coincidental firing. (**A**) Pearson correlation, (**B**) cosine similarity, (**C**) Hamming distance, (**D**) SPIKE similarity, (**E**) scaling factor, (**F**) mutual information, (**G**) transfer entropy, and (**H**) redundancy reduction are applied to spike trains generated from $p(x;\eta_{1},\eta_{2},0)$ and $p(x;\eta_{1},\eta_{2},d\theta )$, over different values of $\eta_{1}$ and $\eta_{2}$ shown by different colored plots (with the constraint $\eta_{1}+\eta_{2}=1$), and averaged over 10 samples. The error bars show the standard error of the mean.

Figure 2 shows the average values of existing indices computed on sampled pattern pairs drawn from control and comparison distributions, $p(x;\eta_{1},\eta_{2},0)$ and $p(x;\eta_{1},\eta_{2},d\theta )$, described in Section 2.3.1. Note that these plots are not symmetrical around dθ=0 since there is an upper limit for how much the two neurons can be correlated, given that $\eta_{2}\leq \eta_{1}$. Recall from Equation (6) that the distance between these distributions is proportional to dθ. As such, an appropriate index of pattern separation should be sensitive to the increase in dissimilarity between patterns from $p(x;\eta_{1},\eta_{2},0)$ and $p(x;\eta_{1},\eta_{2},d\theta )$, as the magnitude of dθ grows larger.

However, Figure 2 shows that, with the exception of mutual information and transfer entropy, existing indices are insensitive to dθ. No index among the Pearson correlation (Figure 2A), cosine similarity (Figure 2B), Hamming distance (Figure 2C), SPIKE similarity (Figure 2D), and scaling factor (Figure 2E) displays changes with dθ at all. The lack of change in the scaling factor is most unsurprising since this index is only designed to measure the norm of a vector; in this case, the norm of a pattern is equivalent to the sum of the number of times each neuron fires, which remains fixed throughout the experiment. There does not appear to be a change in transfer entropy (Figure 2G) from systems whose two neurons fire independently, to those with anti-correlated neurons (i.e., when dθ<0); only when the two neurons became strongly correlated (as dθ became sufficiently large when $\eta_{1}=\eta_{2}=0.5$) does transfer entropy decrease. The amount of redundancy reduction (Figure 2H) appears to only vary insignificantly around 0, no matter what the value of dθ is.

Mutual information (Figure 2F) is the only index that varies with dθ, though the trend appears to be non-monotonic. As the systems become more and more anti-correlated (i.e., as dθ<0 grew smaller), the mutual information between patterns where the neurons’ firing rates are uneven (i.e., when $|\eta_{1}-\eta_{2}|$ is large) are decreased but increased for patterns whose neurons fire more evenly. On the other hand, it always seems to decrease as the neurons become more correlated (i.e., when dθ>0 grows larger).

### 4.2. Patterns with Dissimilar Firing Rates in Neuron 2

**Figure 3:**
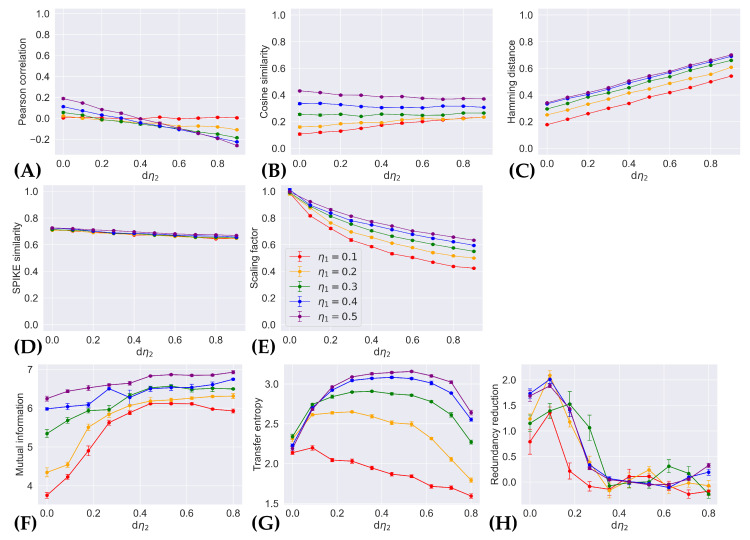
Measurements of existing pattern separation indices versus change in the rate of coincidental firing. (**A**) Pearson correlation, (**B**) cosine similarity, (**C**) Hamming distance, (**D**) SPIKE similarity, (**E**) scaling factor, (**F**) mutual information, (**G**) transfer entropy, and (**H**) redundancy reduction are applied to spike trains generated from $p(x;\eta_{1},0.1,0)$ and $p(x;0.1,0.1+d\eta_{2},0)$, over different values of $\eta_{1}$ shown by different colored plots, and averaged over 10 samples. The error bars show the standard error of the mean.

Figure 3 shows the average values of existing indices computed on sampled pattern pairs drawn from the control and comparison distributions, $p(x;\eta_{1},0.1,0)$ and $p(x;\eta_{1},0.1+d\eta_{2},0)$, where the firing rate of neuron 1 remains fixed between the two distributions, and the firing rate of neuron 2 is increased (see Section 2.3.2 for more detail). Recall that the distance between these distributions is proportional to $d\eta_{2}$. As such, an appropriate index of pattern separation should be sensitive to the increase in dissimilarity between patterns from $p(x;\eta_{1},0.1,0)$ and $p(x;\eta_{1},0.1+d\eta_{2},0)$, as the magnitude of $d\eta_{2}$ grows larger.

Our results complement that of Madar et al. [29] and give another demonstration of how different indices could lead to different interpretations of pattern separation. Pearson correlation (Figure 3A) is less sensitive to neuron 2’s increase in activity if neuron 1 fires sparsely, while the opposite is true for cosine similarity (Figure 3B). The Hamming distance (Figure 3C) appears to increase linearly with $d\eta_{2}$, while the SPIKE similarity (Figure 3D) and scaling factor (Figure 3E) decrease. Among the non-information-theoretic measures, the scaling factor appears to be the only index to change non-linearly with $d\eta_{2}$. Unlike their non-information-theoretic counterparts, the indices mutual information (Figure 3F), transfer entropy (Figure 3G), and redundancy reduction (Figure 3H) all exhibit non-monotonic behavior with increasing $d\eta_{2}$.

## 5. Discussion

We have conceptualized pattern separation in information-geometric terms, and instantiated a simple, highly controlled example using a two-neuron system with a closed-form probability law. This allowed us to test the behavior of commonly used pattern separation measures under tightly controlled circumstances. Specifically, our simple model can generate separated patterns by either (A) changing *which* neurons are active (i.e., altering each neuron’s marginal firing rates), or (B) changing the correlation of activity between the two neurons.

Our work builds on previous evaluations of pattern separation indices. Madar et al. [29] evaluated commonly used indices using recordings of input and output spike trains of single hippocampal neurons, and showed that different indices could yield different results and interpretations. Our results further support their observation. We also evaluated different pattern separation indices for specific weaknesses, much like Bird et al. [30] had done, but with a clearer mathematical formulation of pattern separation. In doing so, we show that existing pattern separation measures, including the information-theoretic measures proposed by Bird et al. [30], fail to recognize pattern separation when it is implemented by changing neuronal co-activation rates, rather than individual neurons’ marginal firing rates. This limitation of the existing measures may restrict the study of pattern separation in circuits in which pattern separation occurs via a temporal coding mechanism [22,29].

Our information-geometric formulation of pattern separation can be extended to include more complex neural dynamics. The two-neuron model serves as a simple example that allows us to isolate the effects of neuronal co-activation. Clearly, it omits many detailed behaviors of real neural systems. For example, the distribution $p(x;\eta_{1},\eta_{2},\theta )$ describes neural systems in equilibrium (i.e., the probability of neural activity is time independent), and different families of distributions (i.e., a different statistical manifolds) could be employed to introduce the temporal structure of spikes or refractoriness after a spike [47,48]. Similarly, different sources of background noise could potentially be investigated through more complex distributions. Noise is inherently considered in our simulations, where spike trains are sampled from categorical distributions, with distinct sets of parameters (e.g., the probability that a neuron fires) presumed to store and convey neural information. Two distinct spike trains sampled from the same distribution, therefore, carry the same information despite the spike trains not being identical. The indices of pattern separation evaluated in this study are affected by background noise as demonstrated by Bird et al., and are reflected in our results by the non-zero error bars (which show the standard error of the mean) in Figure 2 and Figure 3.

Our results encourage a search for new pattern separation indices. Neuronal co-activation, as implemented in our study, may occur due to a third neuron in a separate layer that projects into a larger layer. This neuronal circuit architecture is consistent with expansion re-coding [9,49]. Additionally, increasing the level of connectivity within a neuronal population, such as dentate gyrus circuits with high degrees of mossy-fiber sprouting, may also lead to increased neuronal co-activation rates. Mossy-fiber sprouting is commonly studied in epilepsy, for which pattern separation deficits have been reported in silico [33]. To sufficiently study pattern separation within these systems, we argue that new measures capable of capturing the effects of neuronal co-activation rates must be applied. Inspiration for these measures might be taken from various existing techniques designed for the in-depth analysis of spatiotemporal patterns, such as generating surrogate data (applicable when an analytical approach is not feasible) [50] or convolving the cross-correlation histogram [51] to detect precise, higher-order temporal correlations between spike trains. In addition to detecting patterns from spike trains, the pattern grouping algorithm [52,53] (built on the pattern detection algorithm from Abeles and Gerstein [16]), is also capable of evaluating these patterns’ statistical significance. These methods may provide more robust ways to capture the various neural features (neuronal co-activation rates, in particular) that are relevant to pattern separation. Another such measure could, of course, be the distance between probability distributions underlying different neural patterns. For high-dimensional systems (i.e., models with many neurons and/or parameters), however, we would require efficient methods by which geodesic distances between distributions can be estimated. This requires efficiently estimating Fisher information matrices *G*, whose dimension increases exponentially with the number of neurons even in simple models [32]. However, progress in this area may help us better understand the computational nature of pattern separation as well as neural systems that perform pattern separation.

## Figures and Tables

**Table 1:** Existing indices of pattern separation evaluated in this study.

Symbol	Description	Equation
$d_{\rho }\left(x^{\left(1\right)},x^{\left(2\right)}\right)$	Pearson correlation	(10)
$d_{\theta }\left(x^{\left(1\right)},x^{\left(2\right)}\right)$	Cosine similarity	(11)
$d_{\phi }\left(x^{\left(1\right)},x^{\left(2\right)}\right)$	Scaling factor	(12)
$d_{\eta }\left(x^{\left(1\right)},x^{\left(2\right)}\right)$	Hamming distance	(13)
$d_{S}\left(T^{\left(1\right)},T^{\left(2\right)}\right)$	SPIKE similarity	(14)
${\hat{d}}_{M}\left(T^{\left(1\right)},T^{\left(2\right)}\right)$	Estimated mutual information	(15)
$d_{T}\left[T^{\left(1\right)}:T^{\left(2\right)}\right]$	Transfer entropy	(16)
$d_{R}\left(T^{\left(1\right)},T^{\left(2\right)}\right)$	Relative redundancy reduction	(17)

## Data Availability

Data and code can be made available by request.
